# The ability of diaphragmatic excursion after extubation to predict the need for resumption of ventilatory support in critically ill surgical patients

**DOI:** 10.1007/s00540-024-03442-1

**Published:** 2025-01-05

**Authors:** Ahmed Hasanin, Mina A. Helmy, Ayman Aziz, Maha Mostafa, Mostafa Alrahmany, Mamdouh M. Elshal, Walid Hamimy, Ahmed Lotfy

**Affiliations:** 1 Department of Anesthesia and Critical Care Medicine, Cairo University, Cairo, Egypt; 2 Department of Anesthesia and Critical Care Medicine, National Cancer Institute, Cairo University, Cairo, Egypt

**Keywords:** Weaning, Mechanical ventilation, Reintubation, Diaphragmatic excursion, Critically ill, Ultrasound

## Abstract

**Background:**

This study evaluated the ability of diaphragmatic excursion (DE), measured 2 h after extubation, to predict the need for resumption of ventilatory support within 48 h in surgical critically ill patients.

**Methods:**

This prospective observational study included adult surgical critically ill patients intubated for > 24 h and extubated after a successful spontaneous breathing trial. Sonographic measurement of the DE was performed 2 h after extubation. Patients were followed up for 48 h after extubation and were divided into reintubation group and successful weaning group. The primary outcome was DE’s ability to predict the need for resumption of ventilatory support using the area under receiver characteristic curve (AUC) analysis.

**Results:**

Data from 70 patients were analyzed and 25/70 (36%) patients needed reintubation. DE was lower in the reintubation group than the successful weaning group. The AUC (95% confidence interval) for the ability of DE to predict the need for resumption of ventilatory support was 0.98(0.92–1.00) and 0.97(0.89–1.00) for the right and left side, respectively. At cutoff values of 20.8 and 19.8 mm, the right and left DE had positive predictive values of 92% and 88% and negative predictive values of 96% and 93%, respectively.

**Conclusion:**

Among surgical critically ill patients undergoing weaning from invasive mechanical ventilation, DE obtained 2h after extubation is an accurate predictor for the need for resumption of ventilatory support. Diaphragmatic excursion < 20–21 mm could predict the need for resumption of ventilatory support with a positive predictive value of 88–92% and negative predictive value of 93–96%.

## Introduction

Discontinuation of invasive mechanical ventilation is a critical and frequent challenge in intensive care units (ICUs). Reintubation is commonly associated with serious complications such as arrhythmia, hypoxia, nosocomial pneumonia and increased mortality [1–3]. Early prediction for the need of reintubation during a spontaneous breathing trial (SBT) is aimed to avoid the complications of reintubation and many parameters were evaluated for this purpose [4]. However, despite the presence of several parameters and guidelines for weaning, there are still patients who fail after extubation, and early detection of these patients could enhance their management and alert the intensivist for diagnostic and therapeutic interventions which might avoid reintubation.

The diaphragm is the main respiratory muscle representing more than 70% of the respiratory pump [5]. Diaphragmatic dysfunction plays a major role in difficult weaning from mechanical ventilation [6]. The use of ultrasound in the evaluation of diaphragmatic function allowed its implementation in daily practice for being feasible at the bedside. Ultrasound-derived diaphragm indices showed good accuracy in predicting successful weaning from invasive mechanical ventilation [7, 8] and were able to predict failure of non-invasive ventilation [9]. However, no data, to the best of our knowledge, investigated the role of diaphragmatic excursion (DE) after extubation in predicting the need for resumption of ventilatory support in surgical critically ill patients. This study aims to evaluate the accuracy of DE, 2 h after extubation, in the prediction of the need for the need for resumption of ventilatory support.

## Methods

This prospective observational study was conducted at the surgical ICU in a University Hospital, between September 2022 and October 2023 after the Research Ethics Committee approval (MD-171–2022). Written informed consent was obtained from the patient’s next-of-kin before enrollment.

This study included adult (> 18 years) surgical critically ill patients who were intubated for 24 h and extubated after a successful SBT.

Exclusion criteria were diaphragmatic paralysis, neuromuscular diseases, preexisting lung pathology (e.g. tumor), and pregnancy.

Identifying the patient’s eligibility for weaning and extubation was according to the attending intensivist discretion and according to the local protocol which includes PaO_2_/fraction of inspired oxygen (FiO2) > 200, requiring positive end-expiratory pressure [PEEP] of 8 cmH_2_O and FiO_2_ ≤ 0.4, hemodynamically stable or on minimal vasopressor dose, adequate cough, and Glasgow coma scale > 8. [2] Patients fulfilling criteria for weaning from mechanical ventilation underwent an SBT for 30 min. The SBT was performed using a pressure support of 5 cmH_2_O, FiO_2_ 0.4, and PEEP 5 cmH_2_O. The patient was extubated if the SBT was considered successful (respiratory rate was < 35 breath/min, peripheral oxygen saturation (SpO2) > 90% or PaO_2_ > 60 mmHg on FiO2 < 40%, hemodynamically stable, and no sign of increased work of breathing in the form of nasal flaring, use of accessory muscle, intercostal retraction and paradoxical movement of the ribcage and abdomen) [10]. Patients were excluded if they failed to pass the SBT.

### Ultrasound measurement of DE

The ultrasound assessment was in accordance with the latest consensus on diaphragm ultrasonography in critically ill (EXODUS) [11] and previous literature [7, 8].

Ultrasound assessment for diaphragmatic function was done 2 h after extubation by an expert operator (AA) who was blinded to other patient’s clinical data and had no further involvement in the study. Diaphragmatic movement was measured using a curvilinear transducer (3–5 MHz**)** during normal tidal breathing. The transducer was placed over one of the lower intercostal spaces in the right anterior axillary line for the right diaphragm and the left midaxillary line for the left diaphragm. The transducer was directed toward the diaphragmatic dome at an angle of not < 70°. The M-mode tracing was applied, and the amplitude of the excursion was measured on the vertical axis of the tracing between inspiration and expiration [7, 8]. The attending intensivist was blinded to the data of the ultrasound examination.

The patients were followed up for 48 h to assess the need for reintubation. Patients received supplemental simple oxygen therapy after extubation, and the oxygen flow was adjusted to maintain the SpO_2_ at 89–92%. The extubation was considered a failure if the respiratory rate was > 35 breath/min, SpO_2_ < 90% despite the use of supplemental oxygen, or if there were signs of increased work of breathing in (e.g., the use of accessory muscle, intercostal retraction and paradoxical movement of the ribcage and abdomen). Failure of extubation was also considered if there was significant acidosis or hemodynamic instability [12]. The failure of extubation was managed by invasive mechanical ventilation.

The following data were collected: demographic data, Acute Physiology and Chronic Health Evaluation (APACHE) II score, primary pathology, main cause of mechanical ventilation, days of mechanical ventilation before the weaning trial, time to reintubation, arterial blood gases and rapid shallow breathing index at the end of the SBT, hemodynamic data and respiratory rate 2 h after extubation.

The primary outcome was the ability of DE to predict the need for resumption of ventilatory support 48 h after extubation. Secondary outcomes were identifying risk factors of the need for resumption of ventilatory support and comparing the ability of DE in predicting the need for resumption of ventilatory support to that of other risk factors, namely respiratory rate and rapid shallow breathing index. We also calculated the respiratory rate/DE index by dividing the respiratory rate by the right DE and evaluated its ability to predict the need for resumption of ventilatory support.

Sample size:

Sample size was calculated using MedCalc Software V 18 (Ostend, Belgium). Assuming that the incidence of reintubation was 20% [10, 13], a minimum sample of 65 patients (at least 13 reintubation) was needed to detect an area under receiver operating characteristics (AUC) of 0.75, with a study power of 80%, alpha error of 0.05, and the null hypothesis was set at 0.5.

### Statistical analysis

Statistical analysis was performed using Statistical Package for Social Science (SPSS) version 26 for Microsoft (IBM Corp., NY, USA) and MedCalc Software. Patients were categorized into two groups: successful weaning and reintubation groups. The distribution of continuous data was checked using the Shapiro–Wilk test. Continuous data are reported as mean ± standard deviation or median (quartile) as appropriate. Normally distributed data were analyzed using the Student t test and skewed data were analyzed using the Mann–Whitney U test. Categorical data are summarized as counts (percentages) and were analyzed using the Chi-squared or Fisher’s exact test as appropriate. The ability of DE, respiratory rate, and rapid shallow breathing index to predict the need for resumption of ventilatory support was assessed using the AUC analysis. The best cut-off value was calculated using Youden’s index, and sensitivity, specificity, positive and negative predictive values of the best cut-off value are presented. Comparison of the AUCs was performed using the DeLonge test. We conducted a multivariate logistic regression analysis to identify independent risk factors of the need for resumption of ventilatory support using the forward selection method. The forward selection method was chosen due to the small number of positive cases relative to number of variables, aiming to minimize the risk of model overfitting. The model included the known risk factors of reintubation (age, APACHE II score, and rapid shallow breathing index, PaO_2_/FiO_2_ ratio, and PaCO_2_ at the end of SBT) [13] in addition to the primary pathology, respiratory rate and mean DE (calculated as the average of right and left DE) 2 h after extubation. The analysis started with no predictors in the model and sequentially added variables based on their statistical significance, evaluated using likelihood-ratio tests. The final model identified two variables: mean DE and respiratory rate, and their odds ratio and 95% confidence interval are reported. A *P*-value < 0.05 was considered statistically significant.

## Results

**Fig. 1:**
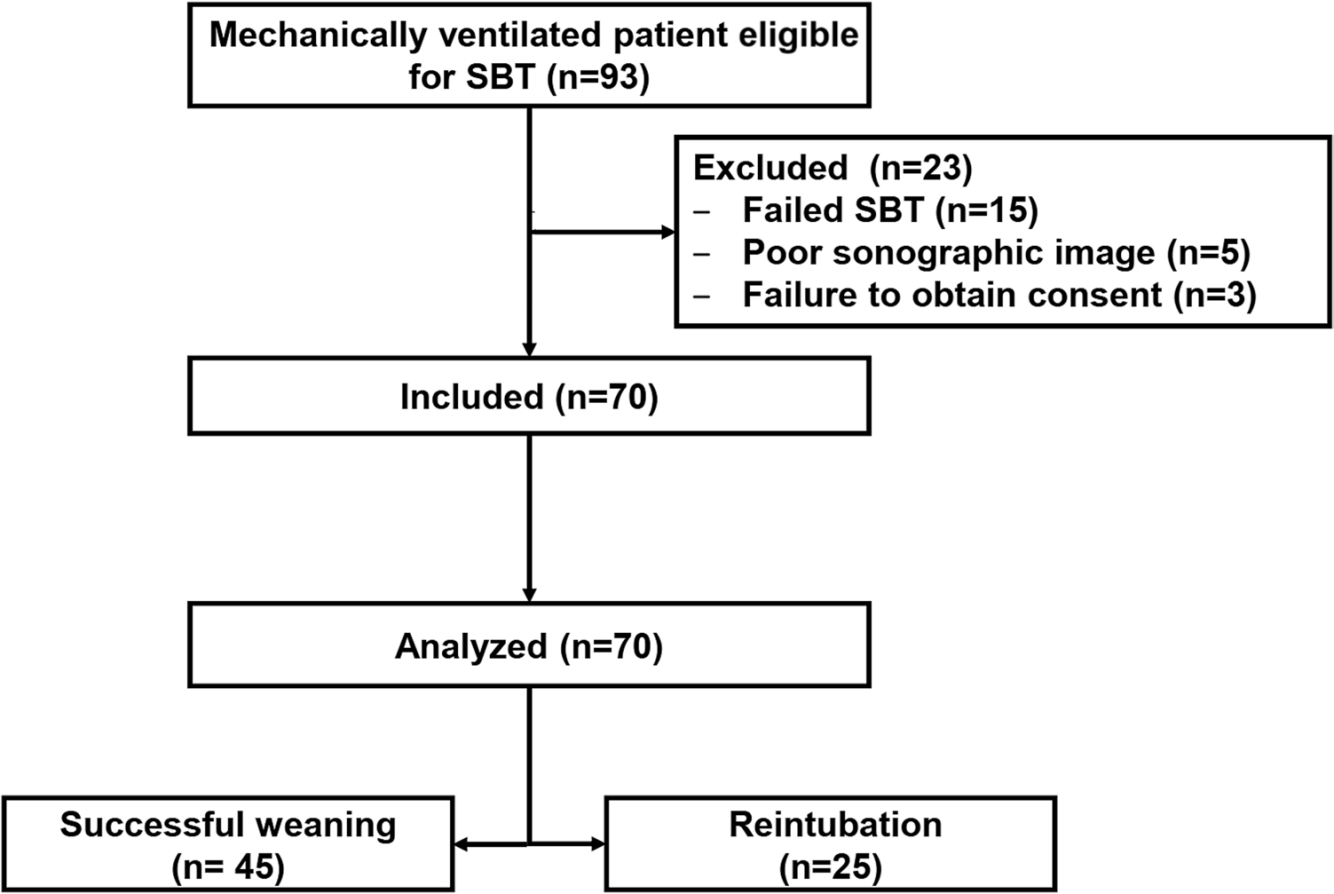
Patients’ enrollment flowchart

Ninety-three patients were screened for eligibility and 23 patients were excluded for either failing the SBT (*n* = 15), poor sonographic image (*n* = 5) or failure to obtain consent (*n* = 3). Seventy patients were included in the final analysis. The majority of the included patients were admitted immediately after emergency surgical procedures (55/70 [79%]). The number of patients needing reintubation was 25/70 (36%) and the median (quartiles) time to reintubation was 10 (7, 17) h from the extubation. (Fig. 1) We did not use preventive noninvasive mechanical ventilation after extubation since none of the participants was intubated due to exacerbation of chronic obstructive pulmonary disease (COPD) nor congestive heart failure [14]. One patient had hypercapnia at the end of the SBT (PaCO_2_ > 45 mmH_2_O); however, this patient was admitted after surgical repair of perforated duodenum and noninvasive mechanical ventilation would be inappropriate in such a patient. Most of our patients had nasogastric tubes which preclude the use of noninvasive respiratory support.

Baseline data and the cause of mechanical ventilation are presented in Table 1. The rapid shallow breathing index was higher while the PaO_2_/FiO_2_ ratio and PaCO_2_ were lower in the reintubation group than the successful weaning group. (Table 1).

**Table 1:** Demographic, clinical and laboratory data. Data are presented as mean ± standard deviation, median (quartiles), and frequency (%)

	Successful weaning group (*n* = 45)	Reintubation group (*n* = 25)	*P*-value
Age (years)	50 (36, 59)	58 (40, 66)	0.102
Male sex	30 (67%)	18 (72%)	0.645
APACHE II score	11 (7, 15)	10 (6, 14)	0.722
Primary pathology			0.328
Soft tissue infection	15 (33%)	6 (24%)	
Pneumonia	8 (18%)	1 (4%)	
Intestinal obstruction	4 (9%)	6 (24%)	
Perforated viscus	4 (9%)	5 (20%)	
Trauma	4 (9%)	2 (8%)	
Burn	3 (7%)	3 (12%)	
Pancreatitis	2 (4%)	0 (0%)	
Stroke	2 (4%)	0 (0%)	
Atrial fibrillation	1 (2%)	0 (0%)	
Chronic limb ischemia	1 (2%)	2 (8%)	
Soft tissue infection	15 (33%)	6 (24%)	
Intraabdominal abscess	1 (2%)	0 (0%)	
Cause of mechanical ventilation			0.253
Hemodynamic instability	30 (67%)	16 (64%)	
Respiratory failure	7 (16%)	7 (28%)	
Disturbed conscious level	5 (11%)	0 (0%)	
Severe metabolic acidosis	3 (7%)	2 (8%)	
Parameters at the end of SBT			
Rapid shallow breathing index	40 (28, 60)	65 (54, 75)	0.001
PaO_2_/FiO_2_ ratio	330 (300, 388)	290 (255, 317)	< 0.001
PaCO_2_ (mmHg)	37 ± 5	33 ± 7	0.005
pH	7.43 ± 0.07	7.44 ± 0.08	0.578
HCO_3_ (mmol/L)	25 ± 4	23 ± 4	0.139
Days of mechanical ventilation	3 (2, 5)	4 (3, 6)	0.062

*APACHE* acute physiology and chronic health evaluation, *SBT* spontaneous breathing trial

The heart rate and respiratory rate were higher in the reintubation group than in the successful weaning group. On the other hand, the DE was lower in the reintubation group than in the successful weaning group. (Table 2).

**Table 2:** Clinical data and ultrasonographic measurements 2 h after extubation. Data presented as mean ± standard deviation and median (quartiles)

	Successful weaning group (*n* = 45)	Reintubation group (*n* = 25)	*P*-value
Clinical data			
Heart rate (beat/min)	94 ± 14	101 ± 13	0.030
Mean blood pressure (mmHg)	81 (78, 87)	80 (70, 93)	0.806
Respiratory rate (breath/min)	20 (17, 22)	28 (25, 29)	< 0.001
Ultrasound measurement			
Right DE (mm)	26 (23, 36)	18 (15, 20)	< 0.001
Left DE (mm)	25 (21, 36)	17 (15, 19)	< 0.001
Mean DE (mm)	25 (22, 37)	17 (15, 19)	< 0.001

*DE* diaphragmatic excursion

**Fig. 2:**
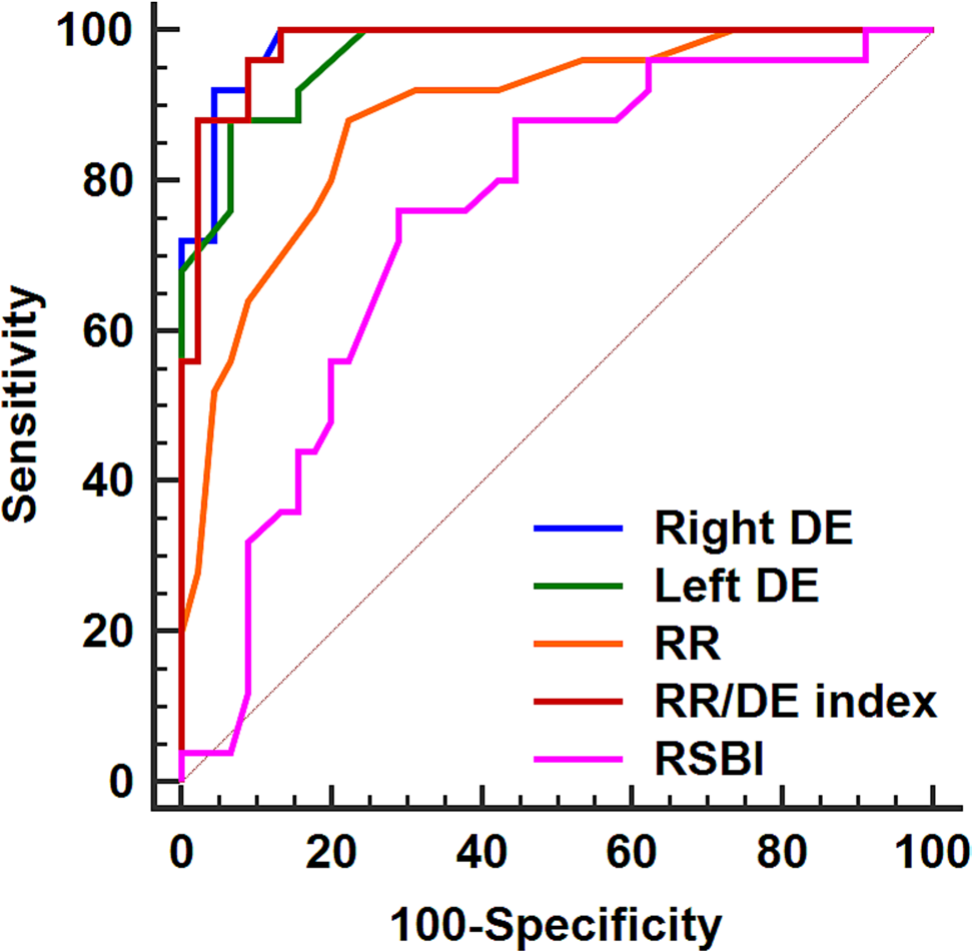
Receiver operating characteristic curves for the ability to predict the need for resumption of ventilatory support. *DE* diaphragmatic excursion, *RR* respiratory rate, *RSBI* rapid shallow breathing index

The AUC (95% confidence interval) for the ability of DE to predict the need for resumption of mechanical ventilation was 0.98 (0.92–1.00) for the right side and was 0.97 (0.89–1.00) for the left side. (Table 3) At a cutoff value of 20.8 and 19.8 mm, the right and left DE had a negative predictive value of 96% and 93%, respectively. (Table 3) (Fig. 2) Furthermore, the right DE AUC was higher than that of the respiratory rate, P-value 0.017. (Table 3) (Fig. 2) The AUC for both right and left DE were higher than that of the rapid shallow breathing index, P-value < 0.001 for both comparisons. (Table 3) (Fig. 2).

**Table 3:** The AUC analysis for the ability to predict the need for resumption of ventilatory support

	AUC (95% CI)	Sensitivity % (95% CI)	Specificity% (95% CI)	PPV% (95% CI)	NPV% (95% CI)	Cutoff value
Right DE (mm)	0.98 (0.92–1.00)*†	92 (74–99)	96 (85–100)	92 (74–99)	96 (85–100)	≤ 20.8
Left DE (mm)	0.97 (0.89–1.00)†	88 (69–98)	83 (82–99)	88 (69–98)	93 (82–99)	≤ 19.8
RR (breath/min)	0.89 (0.79–0.95)†	88 (69–98)	78 (63–89)	69 (50–84)	92 (79–98)	> 22
RR/DE index	0.98(0.91–1.00) *†	96(80–100)	91(79–98)	86(67–96)	97(87–100)	> 1.11
RSBI	0.75(0.63–0.84)	76(55–91)	71(56–84)	59(41–76)	84(69–94)	> 55

^*^ Denotes significance in relation to the RR, †denotes significance in relation to the RSBI

*AUC* area under receiver operating characteristic curve, *CI* confidence interval, *DE* diaphragmatic excursion, *NPV* negative predictive value, *PPV* positive predictive value, *RR* respiratory rate, *RSBI* rapid shallow breathing index

The AUC for respiratory rate/DE index was not significantly different from that of the DE but was significantly higher than that of the respiratory rate and rapid shallow breathing index. (Table 3) (Fig. 2).

The DE was the only significant risk factor for the need for resumption of ventilatory support in a multivariate analysis model including other known risk factors. (Table 4).

**Table 4:** Multivariate analysis for risk factors for the need for resumption of ventilatory support

	Odds ratio (95% Confidence interval)	*P*-value
Mean DE	0.16 (0.03 – 0.79)	0.024
Respiratory rate	1.48 (0.93 – 2.33)	0.096

Multivariate logistic regression model results using the forward selection method. Variables, namely age, APACHE II score, rapid shallow breathing index, PaO_2_/FiO_2_ ratio, PaCO_2_, primary pathology, respiratory rate and mean DE, were included in the model sequentially based on their statistical significance, as determined by likelihood-ratio tests. The final model included mean DE and respiratory rate. *APACHE* acute physiology and chronic health evaluation, *DE* diaphragmatic excursion

## Discussion

We report that DE, obtained two hours after extubation, can accurately predict the need for resumption of ventilatory support within 48 h. The diaphragm is the key inspiratory muscle that contributes to approximately 70% of the normal tidal volume [15] and there is a positive correlation between DE and lung inspiratory volumes making this measure an accurate indicator of respiratory muscle strength and function [16, 17].

Diaphragmatic dysfunction is now recognized as an important measure for readiness-to-wean among patient receiving invasive mechanical ventilation [16]. For being accurate and applicable at the bedside, ultrasonographic evaluation of DE had gained an increasing role in critically ill patients before intubation and during weaning [16, 18]. The majority of previous studies evaluated diaphragmatic ultrasound during the SBT [16, 18]; therefore, a considerable proportion of patients in previous studies were considered “failure” without being actually extubated. The current study has two important differences from the previous literature. First, the focus on resumption of ventilatory support after extubation, which carries more cardiovascular and airway complications than failure during the SBT. Second, the evaluation of DE after extubation as an early measure for respiratory failure. Furthermore, extubated patients are expected to have better diaphragmatic function than non-extubated patients, and consequently different cutoff value, indicating the need for separate assessment of DE after extubation.

In line with our findings, one recent study by Eksombatchi et al. [19] evaluated the DE after extubation and showed that DE can predict reintubation; However, the authors reported a lower accuracy (AUC: 0.73) than ours (AUC: 0.98). This difference could be related to the different population. Eksombatchi et al. [19] included mostly medical patients while our study included emergency surgical patients. Dres et al. [20] had reported that diaphragmatic thickening fraction, another measure of diaphragmatic function, measured after extubation can predict reintubation. However, the main difference between our study and Dres et al. [20] is that we evaluated DE. Diaphragmatic excursion has two advantages over diaphragmatic thickening fraction. First, being easier to measure [11]. Second, the average diaphragmatic thickening fraction values are about 1.5–2.0 mm; therefore, any slight operator-dependent variation would affect measurement [16].

We report that a DE cutoff value ≈20 mm can discriminate patients at risk for the need for resumption of ventilatory support, which is close to cutoff values reported in most studies in non-intubated patients [7, 21]. Studies that evaluated DE before extubation reported lower values (≈10–13 mm) [8] [22]. This difference in the cutoff values seems logical because patients included in our study had already passed the SBT successfully and therefore, they would likely have better respiratory muscle function, and consequently DE, than patients who did not pass the SBT and some of them were not extubated. Furthermore, the diaphragmatic excursion is affected by the use of positive pressure (augment the DE) and PEEP (reduces the magnitude of DE) [11]; therefore, depending on the methods of SBT, the magnitude of DE would vary, affecting the accuracy as well as the cut-off value. Eksombatchi et al. [19] reported a lower cutoff value than ours (10.5 mm) despite including patients after extubation. However, Eksombatchi et al. [19] assessed DE during noninvasive mechanical ventilation in some patients who were deemed high-risk for reintubation, and this might explain the lower cutoff value.

During the first few hours after extubation, the vital signs, namely heart rate and respiratory rate, were modestly higher in patients who were reintubated thereafter; however, the multivariate analysis showed that DE was the only significant risk factor. This finding highlights the value of DE in the early detection of respiratory failure. However, the lack of significance for other variables might be attributed to the low number of positive events relative to the number of variables included in the model, which limited the study's power to detect associations. Combining the respiratory rate and DE into a single index did not significantly improve its accuracy but it improved the DE sensitivity to predict the need for resumption of ventilatory support. Eksombatchai et al. [19] also evaluated the combination of respiratory rate with both diaphragmatic function measures namely the DE and diaphragmatic thickening fraction. The authors reported that the combination of respiratory rate and diaphragmatic thickening fraction had improved the sensitivity and specificity of the test with fair accuracy (AUC: 0.76) in comparison to other measures [19].

Reintubation is a critical incident that represents an independent risk factor for mortality in the ICU [3] [23, 24]. Predicting weaning failure is usually performed during the SBT before extubation; however, it is still important to have a post-extubation evaluation strategy because there is still a proportion of patients who are reintubated, despite passing a successful guideline-based SBT [25, 26]. Early detection of patients who are at high risk for reintubation would alarm the physician to initiate diagnostic and therapeutic measures for the responsible pathology; performing physiotherapy; and calling for senior help. Early detection of high-risk patients would also boost the early initiation of non-invasive ventilatory support. Several protocols for non-invasive support such as non-invasive positive pressure ventilation and/or high-flow nasal cannula, had been implemented after extubation to decrease the likelihood of reintubation [26–28]. However, it is still unclear which groups of patients would benefit from these protocols [29, 30]. Some reports suggested that prophylactic respiratory support after extubation might be useful to high-risk patients according to pre-extubation measurements [29, 30]. Post-extubation measurements might be helpful in detecting patients who would benefit from prophylactic non-invasive respiratory support. In the post-pandemic era, there is an increased awareness for the importance of rapid clearance of intensive care unit beds. Evacuation of the beds after extubation would enable helping new patients and saving hospital resources. Thus, accurate bedside tools for confirming successful weaning are important. Our results introduce a new and important use for diaphragmatic ultrasound which showed excellent accuracy in predicting the outcomes of patients after extubation. Future larger studies are needed to confirm our findings and validate the cut-off value which identifies high-risk patients before including the DE into weaning protocols.

There are some limitations in the current study. It is a single-center study. The routine use of non-invasive respiratory support after weaning is conditionally recommended in the guidelines [14]. However, we did not use preventive non-invasive support since the criteria for high-risk patients did apply to our participants; none were intubated due to exacerbation of COPD nor congestive heart failure. Furthermore, most of our patients had either recent gastrointestinal surgery, nasogastric tube in place, or facial burn which made the placement of a noninvasive mechanical mask either contraindicated or impractical. The use of a high-flow nasal cannula was limited by its availability in our unit. The patients in this study were strictly emergency surgical who were admitted mainly postoperatively after emergency surgery or due to developing complications secondary to the surgical pathology. None of our patients were admitted after a neurosurgical procedure. Therefore, future studies should focus on other groups of patients such as neurosurgical and medical patients. The reintubation rate in the current study is relatively high due to the complicated surgical nature of our patients. Most of our patients had advanced emergency surgical pathology with a high complication rate because our hospital is the largest tertiary referral hospital in our country [31]. A significant proportion of failures was linked to hemodynamic instability and surgical complications. Previous studies showed similar rates of reintubation in surgical critically ill patients [32, 33]. The high number of positive cases contributed to the high PPV of our cutoff values.

In conclusion, among surgical critically ill patients undergoing weaning from invasive mechanical ventilation, DE two hours after extubation is an accurate predictor for the need for resumption of ventilatory support. Diaphragmatic excursion < 20–21 mm could predict the need for resumption of ventilatory support with positive predictive value of 88–92% and negative predictive value of 93–96%.

## Data Availability

Data are available at the corresponding author upon any reasonable request.
